# Psychological factors associated with changes in physical activity in Dutch people with type 2 diabetes under societal lockdown: A cross‐sectional study

**DOI:** 10.1002/edm2.249

**Published:** 2021-05-05

**Authors:** Hannah Regeer, Emma A. Nieuwenhuijse, Rimke C. Vos, Jessica C. Kiefte‐de Jong, Pepijn van Empelen, Eelco J. P. de Koning, Henk J. G. Bilo, Sasja D. Huisman

**Affiliations:** ^1^ Division of Endocrinology Department of Medicine Leiden University Medical Center Leiden The Netherlands; ^2^ Department of Public Health and Primary Care / LUMC‐Campus The Hague Leiden University Medical Center Den Haag The Netherlands; ^3^ TNO Research Group Child Health Leiden The Netherlands; ^4^ Diabetes Knowledge Centre Isala Zwolle The Netherlands; ^5^ Faculty of Medicine University of Groningen Groningen The Netherlands; ^6^ Department of Internal Medicine University Medical Center Groningen Groningen The Netherlands

**Keywords:** COVID‐19, physical activity behaviour, stress, type 2 diabetes

## Abstract

**Aims:**

To investigate changes in physical activity (PA) and psychological factors during societal lockdown in people with type 2 diabetes.

**Methods:**

A cross‐sectional study among Dutch adults with type 2 diabetes. Data were collected using online questionnaires. A multivariate multinomial logistic regression was performed with change in PA during societal lockdown as outcome and perceived change in stress, anxiety, perceived risk for COVID‐19 infection, emotional well‐being and former PA status as determinants.

**Results:**

Five hundred and sixty seven respondents filled out the questionnaire, 536 were included in the final analysis: mean age of 65.9 ± 7.9 years; mean diabetes duration 13.3 ± 8 years; 54% men; 47% reported no change in PA, 27% became less active and 26% became more active during societal lockdown. Participants who were more likely to become less active were participants who experienced more stress (OR: 2.27; 95% CI 1.25–4.13) or less stress (OR: 2.20; 95% CI 1.03–4.71). Participants who were more likely to become more active were participants who experienced more stress (OR: 2.31; 95% CI 1.25, 4.26). Participants with higher emotional well‐being (OR: 0.98; 95% CI 0.97, 0.99) were less likely to become less active than to report no change in PA.

**Conclusions:**

Changes in PA in people with type 2 diabetes during societal lockdown are associated with changes in psychological factors such as perceived stress and emotional well‐being. People with diabetes and their caregivers should be aware of these possible changes.

## Abstract 2

Physical activity is important for the management of diabetes and is influenced by daily life structures. Physical activity behaviour changed in people with type 2 diabetes during the societal lockdown in times of the COVID‐19 pandemic. Stress and well‐being are associated with change in physical activity.

## 1 INTRODUCTION

The world's population has been physically and socially affected by the pandemic of the SARS‐CoV‐2 (COVID‐19) infection. Mid‐December 2020, over 77 million people were estimated to be infected and over 1.7 million deaths were reported worldwide.^1^ Risk factors for a more severe disease course and mortality are age, obesity, smoking, multimorbidity (including type 2 diabetes), socio‐economic background and ethnicity,^2^, ^3^, ^4^, ^5^

In the Netherlands, national measures to control the COVID‐19 outbreak were taken from 9th March, including strict social distancing, temporarily closing of schools, public buildings, public transport, public events, stores, and sport and wellness centres, and the strong advice to stay and work at home.^6^ These far‐reaching societal lockdown measures had a major impact on private and public life.

Major changes in daily routines impact both mental and physical health.^7^ Recent studies describing the psychological impact of quarantine showed that experiencing quarantine is related to a wide range of stress‐ and mood‐related symptoms, such as depression, anxiety, irritability, poor concentration, insomnia and post‐traumatic stress disorder (PTSD),^8^, ^9^, ^10^, ^11^; Common stressors during quarantine periods are feelings of frustration and boredom, duration of the quarantine, lack of social support, inadequate basic supplies, financial problems, inadequate provision of information and fear of infection.^8^, ^9^

Perceived risk and fear of infection with SARS‐CoV‐2 may increase anxiety levels and stress in people with type 2 diabetes, as studies showed that people with (type 2) diabetes face increased risks of complications and mortality when infected with COVID‐19.^2^, ^3^, ^5^ People with type 2 diabetes who fear infection and perceive their risk of infection with COVID‐19 as higher may put more emphasis on following self‐quarantine restrictions and may be more reluctant to engage in social‐related events, thereby disrupting their daily routines, social life and physical activity (PA). Such disruptions in daily routines and negative emotions are known to negatively influence diabetes self‐management and glycaemic control.^12^, ^13^, ^14^

Daily PA effectively contributes to diabetes self‐management,^15^, ^16^, ^17^ with positive effects on glycaemic control and emotional well‐being.^18^, ^19^, ^20^ This self‐management behaviour is hindered when indoor and (group) outdoor leisure‐time sport activities are prohibited and one is home‐bound, thus also precluding less intensive forms of PA such as walking. Furthermore, increased stress levels are known to inhibit engagement in PA behaviour^21^ and to have a negative impact on overall well‐being. Data on worldwide step count during the COVID‐19 pandemic showed that individual (physical) activity habits change and often decrease under societal lockdown.^22^, ^23^ Furthermore, a Canadian study showed that the direction of change in PA behaviour differed between inactive and active people, where inactive people predominantly became less active and active people became more active during the COVID‐19 pandemic.^24^

In the current study, we investigated changes in PA behaviour and how these changes are associated to perceived change in stress, anxiety, perceived risk of COVID‐19 infection and emotional well‐being during the societal lockdown in people with type 2 diabetes.

## 2 PARTICIPANTS AND METHODS

### 2.1 Population and setting

The current study is an observational cross‐sectional study among people with type 2 diabetes. This is a convenience sample of people with type 2 diabetes who participated in a group‐based diabetes walking intervention during the past 5 years.^25^

All 3127 former participants of the group‐based diabetes walking intervention who had previously agreed and consented to be contacted again for future research purposes, received an information letter and a link to an online questionnaire by e‐mail. Participants were eligible if they were over 18 years old, were able to fill out an online questionnaire and had type 2 diabetes. Participants were excluded if they had co‐morbidity significantly impacting mobility or vitality (e.g. severe/recent cardiac problems, rehabilitation from surgery).

Data collection was in the first week of May 2020, during the national societal lockdown which in the Netherlands started on 9th March. Lockdown measures at that moment were social distancing; closure of schools, day‐care centres, indoor and outdoor sporting facilities, cultural institutions and theatres; working at home if possible; and restriction of public transport use. Individuals were allowed to go outside for groceries, to get some fresh air or to exercise without time limit. Exercising in a group was forbidden. To ensure social distancing, it was not allowed to get together with more than three people and a distance of 1.5 metres had to be kept from each other. If this 1.5 metres was not maintained, people could be charged with a considerable fine and a criminal record.^6^ From the second half of April 2020, there was a gradual decrease in reported COVD‐19 infection rate and—related hospital admissions, and—deaths.^26^ At time of data collection, the established COVID‐19 infection rate was 39,791 people, with a death toll of 4893 people in the Netherlands.^1^ The allowed time to respond to the questionnaire was restricted to 18 days and participants received 2 reminders during this period. It was estimated that participants would need 30 minutes to fill out the questionnaires.

### 2.2 Measures

The online questionnaire included items on demographic information, medical information about diabetes, medication and co‐morbidity, potential COVID‐19 infection, the impact of the societal lockdown on daily routine, changes in PA, former PA status, perceived stress, anxiety for infection and perceived risk of COVID‐19 infection, and current emotional well‐being. All outcome data were self‐reported.

#### 2.2.1 Primary outcome—change in PA behaviour

The primary outcome was the self‐reported change in PA (less active, no change, more active) during the societal lockdown. Participants were asked to indicate whether the number of minutes per week that they actively engaged in leisure‐time activities (such as walking, cycling, gardening) changed during the societal lockdown.

#### 2.2.2 Former PA status

To assess PA status before the societal lockdown, the validated 11‐item Short Questionnaire to Assess Health‐enhancing PA (SQUASH) was used.^27^, ^28^ Outcomes were total moderate‐vigorous PA per week and meeting the national fit norm (>150 minutes moderate‐vigorous PA per week). The fit norm was calculated according to the methods of Wendel‐Vos et al^27^ and was used as indicator of former PA status.

#### 2.2.3 Psychological measures

Perceived change in stress was assessed by asking participants to indicate whether they had experienced changes in overall stress levels (less stress, more stress, no change in stress) during the societal lockdown.

To assess perceived stress level during the COVID‐19 pandemic, the validated 10 item Perceived Stress Scale (PSS) was used (17). Each item assesses the degree to which events are being perceived as being stressful on a five‐point Likert scale (never—almost never—sometimes—fairly often—very often). Four items are stated positively and scored in reverse, before item scores are summed into a total score. Higher total scores represent more perceived stress.

Anxiety for infection was measured with a 10‐point visual analogue scale, asking the participants to rate how anxious they were to get infected with COVID‐19 during the last 6 weeks, with a higher score indicating more anxiety.

To assess perceived risk of COVID‐19 infection, participants were asked to rate on a scale from 1 to 5 their personal risk of infection (1 very unlikely, 5 very likely).

The validated World Health Organization well‐being index (WHO‐5) was used to assess current emotional well‐being.^29^ The five items are assessed on a 6‐point Likert scale ranging from zero to five. The sum of the individual item scores are transformed into a 100‐point scale with lower scores indicating worse well‐being.

#### 2.2.4 Demographic and medical information

Demographic and medical information items included age, sex, educational level, medication use and co‐morbidity, and whether the participants had experienced symptoms or had an actual diagnosis of COVID‐19. Educational level was categorised into low, intermediate and high. Co‐morbidity was grouped into number of co‐morbidities besides diabetes mellitus. Diabetes treatment was defined into three groups: (a) lifestyle only; (b) oral glucose lowering therapy only; and (c) long and/or short acting insulin therapy, with or without oral glucose lowering therapy.

### 2.3 Ethical approval

Ethical approval was obtained by the Medical Ethical committee of the Isala general hospital (Zwolle, the Netherlands; ref nr. 180341). All participants gave written informed consent.

### 2.4 Analysis

Population characteristics and scores on perceived stress, anxiety and perceived risk of COVID‐19 infection, and emotional well‐being were described using descriptive analyses and compared per PA group (decreased PA, unchanged PA, increased PA during societal lockdown) with chi‐square and ANOVA tests. Post hoc comparisons were performed with a Bonferroni‐adjusted significance level of .0056 (.05/9) for the categorical variables and a Bonferroni‐adjusted significance level of .017 (.05/3) for the continuous variables.

A multivariate multinomial logistic regression model was used to analyse the change in PA by perceived change in stress, anxiety, perceived risk for COVID‐19 infection, emotional well‐being and former PA status (compliance to fit norm). Age, sex, educational level as a measure of socio‐economic status, diabetes treatment modality and co‐morbidity were included in the model as covariates. For all analyses, participants with a change in PA (less active or more active) were compared with the participants with no change in PA (reference group) during societal lockdown.

All assumptions for multinomial regression were met. Multi‐collinearity was explicitly tested for the perceived change in stress, well‐being and anxiety and perceived risk of COVID‐19 infection measures using Spearman's correlation coefficient and showed correlations <0.4 (*p* < .001). Significance was set at *p* < .05. Missing data were <10% for all questionnaires; thus, a full sample analysis was performed. All statistical analyses were performed utilizing SPSS‐25.0 software (SPSS Inc.).

## 3 RESULTS

### 3.1 Population characteristics

**FIGURE 1:**
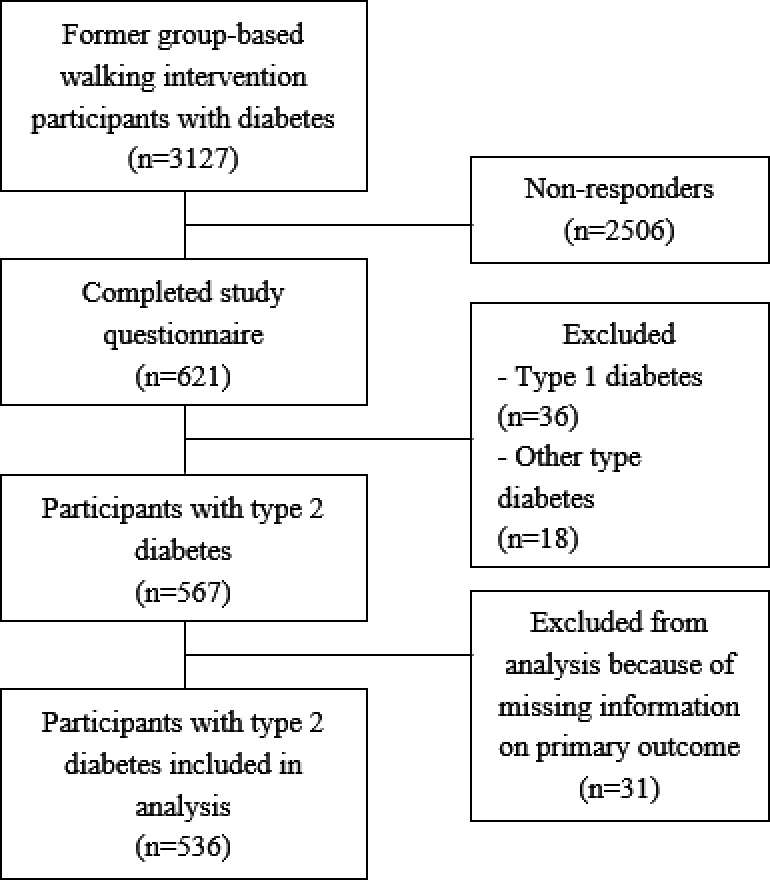
Flow‐chart participant inclusion

Of the 3127 invited, a total of 621 respondents filled out (part of) the questionnaire. Reasons for not completing the questionnaire were unknown for most of the cases. Some people indicated that they had no time for participating or did not want to participate for personal reasons. A total of 567 participants met the inclusion criteria of being diagnosed with T2DM and being over 18 years (see Figure 1 for participation flow diagram). Participants who had missing information on the primary outcome (change in PA behaviour) were excluded from the analysis. Eventually, 536 participants were included in the final analysis (mean age 65.9 ± 7.9 years; 54% men; mean disease duration 13.3 ± 8.1 years). 28.9% of the participants reported a low education level, 43.0% an intermediate education level and 28.1% a high education level. Half of the participants reported 1–2 co‐morbidities, 16% no co‐morbidities and 34% three or more co‐morbidities. Less than 25% reported heart disease, diabetic complications or a chronic lung disease. Most participants did not have COVID‐19 symptoms (86%), and four participants (0.7%) had been tested positive. Before the societal lockdown, the participants reported a median of 420 (IQR 626.3) minutes of moderate to vigorous PA per week, with 56% meeting the fit norm (Table 1). Over 90% was treated for their diabetes in a primary care setting. Most participants avoided to leave their homes or went out only 1–2 times a week (23% and 45%, respectively) during the past 6 weeks prior to filling out the questionnaire. Weight gain was reported by 37% of the participants but most participants reported a stable weight (46%). Self‐reported glycaemic control remained similar in 70% of the participants (Table S1).

**TABLE 1:** Population characteristics of total and per change in PA group in people with type 2 diabetes during COVID‐19 societal lockdown

	Total (*n* = 536)	No change in PA (*n* = 252)	Less active (*n* = 137)	More active (*n* = 147)
Age, years (mean, SD)	65.9 (7.9)	67.5 (7.1)	65.6 (8.1)	63.4 (8.3)
Sex, men, *n* (%)	290 (54.1)	158 (62.7)	65 (47.4)	67 (45.6)
Level of education, *n* (%)				
Low	153 (28.9)	85 (34.3)	35 (25.7)	33 (22.6)
Intermediate	228 (43.0)	102 (41.1)	58 (42.6)	68 (46.6)
High	149 (28.1)	61 (24.6)	43 (31.6)	45 (30.8)
Duration of diabetes, years (mean, SD)	13.3 (8.07)	13.6 (8.44)	14.1 (8.02)	12.1 (7.19)
Diabetes treatment				
Lifestyle advise only	65 (12.3)	30 (12.1)	21 (15.4)	14 (9.6)
Oral antihyperglycaemic therapy only	339 (64.1)	165 (66.8)	83 (61.0)	91 (62.3)
Insulin ± oral antihyperglycaemic therapy	125 (23.6)	52 (21.2)	32 (23.5)	41 (28.1)
Chronic co‐morbidity (*n*, %)^a^				
No co‐morbidity	86 (16.0)	41 (16.3)	11 (8.0)	34 (23.1)
1–2 co‐morbidities	269 (50.2)	138 (54.8)	64 (46.7)	67 (45.6)
>3 co‐morbidities	181 (33.8)	73 (29.0)	62 (45.3)	46 (31.3)
Of which				
Ischaemic heart/ artery disease or cardiac failure	103 (19.2)	50 (19.8)	33 (24.1)	20 (13.6)
Asthma or COPD	77 (14.4)	28 (11.1)	19 (13.9)	30 (20.4)
Diabetic complication^b^	110 (20.5)	51 (20.2)	37 (27.0)	22 (15.0)
COVID−19 complaints during lockdown (*n*, %)				
Yes, tested positive	4 (0.7)	1 (0.4)	1 (0.7)	2 (1.4)
Yes, but tested negative	2 (0.4)	0	2 (1.5)	0
Yes, but not tested	51 (9.5)	19 (7.5)	19 (13.9)	13 (8.8)
No	461 (86.0)	222 (88.1)	112 (81.8)	127 (86.4)
I don't know	18 (3.4)	10 (4.0)	3 (2.2)	5 (3.4)
Meets fit norm *before* societal lockdown^c^ (*N*, %)	299 (56.4)	137 (54.6)	71 (52.6)	91 (63.2)
Minutes moderate‐vigorous intensity physical activity per week *before* societal lockdown (median, IQR)	420 (626.3)	395 (696.8)	345 (515.0)	485 (625.0)

^a^ Co‐morbidities pre‐defined in the following groups: COPD, obesity, ischaemic vascular disease, heart failure, asthma, dyslipidaemia, hypertension, rheumatic disease, arthrosis, kidney failure, polyneuropathy, diabetic ulcer, retinopathy, depression, ‘other’.

^b^ Diabetes complication: retinopathy, nephropathy, polyneuropathy and/or diabetic foot ulcer.

^c^ > 150 min moderate—vigorous physical activity per week.

### 3.2 Change in PA behaviour during the COVID‐19 societal lockdown

A change in PA behaviour was reported by 53% participants during the societal lockdown, of which 27% became more active and 26% became less active.

### 3.3 Psychological status per change in PA group during societal lockdown

No change in stress was reported by 58.9% of the participants, 29.1% reported more stress and 12.0% reported less stress during the societal lockdown (Table 2). This change in stress significantly differed between PA groups (X^2^(4, *N* = 536) = 39.8, *p* < .0001). Participants reporting no change in PA compared with those who became less active or more active, more frequently reported no change in stress (72.4%, vs 53.1% and 40.4%, respectively) and less frequently reported more stress (19.1% vs 32.9% and 43.4%, respectively).

**TABLE 2:** Perceived stress score, perceived change in stress, anxiety for and perceived risk of infection, emotional well‐being, per change in PA group in people with type 2 diabetes during COVID‐19 societal lockdown

	Total (*n* = 536)	No change in PA (*n* = 252)	Less active (*n* = 137)	More active (*n* = 147)
Perceived change in stress				
More stress	153 (29.1)	47 (19.1)^a^	59 (43.4)	47 (32.9)
Less stress	63 (12.0)	21 (8.5)	22 (16.2)	20 (14.0)
No change in stress	309 (58.9)	178 (72.4)^b^	55 (40.4)	76 (53.1)
Perceived Stress Score (mean, SD)	12.98 (6.61)	11.69 (5.95)	15.21 (6.90)^c^	13.11 (6.89)
WHO well‐being index score (mean, SD)	64.9 (23.9)	70.2 (21.6)	53.5 (25.8)^d^	66.7 (22.4)
Anxiety for infection (median, IQR)	4.2 (2.5)	3.9 (2.4)	4.7 (2.5)^e^	4.2 (2.5)
Perceived risk of infection (mean, SD)	2.9 (0.8)	2.8 (0.9)	3.0 (0.7) ^e^	2.9 (0.8)

Note

The *p*‐values represent the comparison between the change in PA groups, using chi‐square tests for categorical variables against a Bonferroni‐adjusted alpha of .0056 (.05/9) and ANOVA's for continuous variables against a Bonferroni‐adjusted alpha of .017 (.05/3).

^a^ Significantly different from increase and decrease in PA group *p* < .0001.

^b^ Significantly different from increase and decrease in PA group *p* < .0001.

^c^ Significantly different from unchanged PA group (*p* < .0001) and increase in PA group (*p* = .007).

^d^ Significantly different from unchanged PA group and increase in PA group (*p* < .0001).

^e^ Significantly different from unchanged PA group on anxiety for infection (*p* = .001) and perceived risk of infection (*p* = .003).

Participants who became less active during societal lockdown compared with those reporting no change in PA or became more active had a higher perceived stress score (mean 15.21 ± SD 6.90, vs 11.69 ± 5.95 and 13.11 ± 6.89, respectively) and a lower emotional well‐being score (mean 53.49 ± SD 25.76, vs 70.18 ± 21.63 and 66.65 ± 22.38, respectively). Perceived stress and emotional well‐being did not differ between people who became more active and those reporting no change in PA.

Both perceived risk of and anxiety for infection were significantly higher for people who became less active (3.01 ± 0.68 vs 4.71 ± 2.45, respectively) than participants reporting no change in PA (2.76 ± 0.85 vs 3.87 ± 2.39) during societal lockdown.

### 3.4 Factors associated with change in PA during societal lockdown

A multivariate multinomial logistic regression was performed to model the relationship between the determinants (perceived change in stress, anxiety for and perceived risk for COVID‐19 infection, emotional well‐being, and former PA status) and change in PA (Table 3). Age, sex, educational level, diabetes treatment and co‐morbidity were included in the model as covariates.

**TABLE 3:** Multivariate multinomial regression model on the factors associated with change in PA in people with type 2 diabetes during COVID‐19 societal lockdown

Factors	Less active versus no change in PA			More active versus no change in PA		
	OR	95% CI	*p* Value	OR	95% CI	*p* Value
Sex (male)	0.69	0.43 to 1.12	.135	0.55	0.34 to 0.88	.012*
Age	0.99	0.96 to 1.03	.699	0.94	0.92 to 0.97	<.0001**
Level of education						
Low	0.51	0.27 to 0.95	.033*	0.45	0.24 to 0.84	.012*
Middle	0.74	0.43 to 1.29	.284	0.89	0.52 to 1.53	.683
High	Ref			Ref		
Diabetes treatment						
Lifestyle only	1.37	0.63 to 2.97	.430	0.60	0.26 to 1.36	.219
Oral antihyperglycaemic therapy only	0.90	0.51 to 1.60	.728	0.71	0.42 to 1.21	.210
Insulin ± oral antihyperglycaemic therapy	Ref			Ref		
Number of co‐morbidities						
None	0.46	0.21 to 1.02	.055	1.40	0.73 to 2.70	.310
1–2	0.64	0.38 to 1.05	.079	0.70	0.41 to 1.18	.181
≥3	Ref			Ref		
Former PA status: compliance to fit norm (yes)	1.07	0.67 to 1.71	.782	1.57	0.99 to 2.50	.056
Perceived change in stress						
More stress	2.27	1.25 to 4.13	.007**	2.31	1.25 to 4.26	.007**
Less stress	2.20	1.03 to 4.71	.042*	1.78	0.82 to 3.85	.143
No change in stress	Ref			Ref		
Anxiety	0.99	0.89 to 1.11	.899	0.96	0.86 to 1.07	.483
Perceived risk of infection	1.18	0.86 to 1.61	.317	1.07	0.78 to 1.45	.685
Emotional well‐being	0.98	0.97 to 0.99	<.001**	1.01	0.99 to 1.02	.230

Note

Reference group = no change in PA. The final model is a significant improvement in fit over a null model. −2Log‐likelihood: final model: 951.9 vs null model: 1070.7 (X^2^(28) = 118.8, *p* < .001). Pearson's chi‐square: X^2^(980) = 1002.42, *p* =0.302; deviance chi‐square: X^2^(980) = 951.91, *p* = .734.

Abbreviations: CI, confidence interval; OR, adjusted odds ratio; Ref, reference category of the factor.

* *p* < .05.

** *p* < .01

Participants who were more likely to become less active were participants who experienced more stress (OR: 2.27; 95% CI 1.25, 4.13) or less stress (OR: 2.20; 95% CI 1.03, 4.71) compared with participants who experienced no change in stress. Participants who were less likely to become less active were participants with a lower education (OR: 0.51; CI 95% 0.27, 0.95) compared with participants with a higher education, and participants with higher scores on emotional well‐being (OR: 0.98; 95% CI 0.97, 0.99). When included in the regression model, the significant association between perceived risk of and anxiety for infection and change in PA became nonsignificant.

Participants who were more likely to become more active were participants who experienced more stress compared with participants who experienced no change in stress (OR: 2.31; 95% CI 1.25, 4.26). Participants who were less likely to become more active were participants with lower education levels compared with participants with higher education levels (OR: 0.45; 95% CI 0.24, 0.84), men compared with women (OR: 0.55; 95% CI 0.34, 0.88), and participants with higher age (OR: 0.94; 95% CI 0.92, 0.97).

## 4 DISCUSSION

The current study showed that in about half of participants (47%) PA behaviour seems to be unaffected by the lockdown measures. However, in line with previous studies on change in PA behaviour during the COVID‐19 pandemic,^13^, ^22^, ^23^, ^24^ our results showed that one in four participants became more active and a similar number became less active. This change in PA was associated with changes in stress and emotional well‐being during the lockdown.

People who experienced more stress were more likely to be less physically active or more physically active. The finding that increased stress levels were related to both increased and decreased PA might be explained by the way people cope with stress. Some people are inhibited in response to stress and tend to display more sedentary behaviour, as other people become more activated and use PA to deal with the stress. Furthermore, the way people react to stress is greatly determined by personality traits.^22^ However, as the current study did not look at coping style and personality no conclusions can be drawn on these aspects.

People who experienced less stress were more likely to be less physically active rather than having no change in PA. This was the case in one out of six participants. For employed individuals, this is possibly explained by less commuting or lower work load because they had to work from home, but might also be explained by personality traits. Furthermore, 40% of the people who became less active reported no change in stress at all. During the lockdown, various activities in daily life, like commuting, sporting activities and social visits, had come to stagnation, which had nothing to do with stress but did cause less activity.

The level of PA before the societal lockdown did not seem to influence the change in PA behaviour during the lockdown in our sample. This is in contrast with the findings of a recent study showing that inactive people predominantly became less active and active people predominantly became more active during the COVID‐19 pandemic.^25^ However, our participants were known to have participated at least once in group‐based walking interventions during the previous 5 years which might have biased this association.

Lower emotional well‐being is associated with lower PA and distress.^30^ Our study showed that people with higher emotional well‐being scores were slightly less likely to become less active during lockdown, suggesting that people with higher well‐being scores might be less susceptible for changes in lifestyle due to lockdown. This is in line with earlier research indicating that people with lower distress are more capable to maintain healthy life style behaviour.^22^ Of course, since we performed a cross‐sectional study, no causality is proven.

Anxiety for and perceived risk of infection were higher for people with type 2 diabetes who were less active than those who reported no change. However, these differences disappeared when other predictors were included in the regression model, while the relationship of change in PA with emotional well‐being and change in stress becomes clearer. A Danish study showed that people with diabetes experienced worries about being at higher risk for a more severe COVID‐19 disease course and being less able to manage their diabetes when infected; these worries were related to increased stress levels,^31^ indicating that higher perceived risk of and anxiety for infection might be one of the reasons for being more stressed during societal lockdown.^31^ However, the increase in experienced stress could also be attributed to other changed factors during societal lockdown, such as relational problems, working at home, children being at home or change in income.^8^, ^9^

The study method had some limitations, including the lack of a measurement of perceived stress and PA performed prior to the COVID‐19 pandemic. Because of this, it was not possible to more objectively assess the change in stress and PA with pre‐post questionnaires; instead, we had to rely on patient‐reported subjective change over a period of 2 months, possibly resulting in a recall bias. Another limitation is the lack of available objective clinical outcomes, such as an HbA1c measurement, which could have provided more information about the (change in) health status of the participants. The nature of the recruitment method and survey instrument may have resulted in selection bias as we might have missed people with an older age, a migration background and low literacy. In future research, the response rate might be improved by for example recruiting through healthcare providers or by offering the option of completing the questionnaire on printed paper rather than online.

The results might be less generalizable to the general population as we included only people with type 2 diabetes that participated in a diabetes walking intervention in the past and might therefore have been more motivated for PA. However, former PA status did not seem to influence the change in PA behaviour in this study. The combination of an older age, lower to intermediate education and a high prevalence of comorbid disorders indicates that our study group is certainly not the most amendable group to engage in PA. These group characteristics correspond to the characteristics of the general diabetes population in the Netherlands, making the results of the current study more generalizable. Given the higher age and high co‐morbidity rate, one would expect this group to be less inclined to become more physically active; however, our results indicated that during the societal lockdown change in PA apparently was not affected by having comorbid conditions.

Because of the cross‐sectional design of the study, we cannot make definite statements about the direction of the association between perceived change in stress and change in PA behaviour. However, looking at the clinical implications of the results, regardless of the direction of the association, we see a group of people experiencing a mental and/or physical decline during the social lockdown and that should be a point of attention within the (online) consultation room.

## 5 CONCLUSION

This study highlights that perceived PA behaviour seems to be differentially affected by the societal lockdown in people with type 2 diabetes and that change in PA is associated with changes in psychological factors such as perceived stress and emotional well‐being.

The results of this study may help create more awareness in people with diabetes, their health care providers and sport professionals about the psychological impact of the lockdown on PA, and the need for monitoring and coaching in maintaining PA—with a focus on changes in stress and emotional well‐being to maintain a healthy lifestyle during a societal crisis.

## CONFLICT OF INTEREST

The authors declare that there is no conflict of interest.

## AUTHOR CONTRIBUTIONS

All authors contributed to the design of the study and developed the methodology. HR and EN performed the data collection, analysed the data, interpreted the results and wrote the manuscript. All authors provided review of the analysis and manuscript and HR and EN revised the manuscript.

## Supporting information

Supplementary Material

## Data Availability

The data that support the findings of this study are available from the corresponding author upon reasonable request.
