# Supplementary material for: Psychological factors associated with changes in physical activity in Dutch people with type 2 diabetes under societal lockdown: A cross‐sectional study
Source: Endocrinol Diabetes Metab. 2021 May 5;4(3):e00249. doi: 10.1002/edm2.249 (PMC8279615; doi:10.1002/edm2.249)
Supplement: Supplementary file 1 — Supplementary Material [file EDM2-4-e00249-s001.docx]

**Supplementary material**

Psychological factors associated with changes in physical activity in Dutch people with type 2 diabetes under societal lockdown: a cross-sectional study

H Regeer^1*^, EA Nieuwenhuijse^2*^, RC Vos^2^, JC Kiefte-de Jong^2^, P van Empelen^3^, EJP de Koning^1^, HJG Bilo^4,5^, SD Huisman^1^

* both authors contributed equally to the article

1. Leiden University Medical Center, Department of Medicine, Division of Endocrinology, Leiden, Netherlands
2. Leiden University Medical Center, Department of Public Health and Primary Care / LUMC-Campus The Hague, Netherlands
3. TNO, Research Group Child Health, Leiden, Netherlands
4. Diabetes Knowledge Centre, Isala, Zwolle, Netherlands
5. Faculty of Medicine, University of Groningen; and Department of Internal Medicine, University Medical Center Groningen, the Netherlands

**Supplementary material**

**Table 1:** Impact of the quarantine period on lifestyle and daily life

| Table 1 Impact of the quarantine period on lifestyle and daily life in people with type 2 diabetes | N=567 |
| --- | --- |
|  |  |
| Change in ability to regulate glucose, N (%) | *N*=565 |
| *Yes, much easier* | 26 (4.6) |
| *Yes, somewhat easier* | 28 (5.0) |
| *No, my glycaemic control remained  the same* | 398 (70.4) |
| *Yes, somewhat more difficult* | 83 (14.7) |
| *Yes, much more difficult* | 30 (5.3) |
| Change in insulin use? N (%) | *N=*227 |
| *Yes, I use much less insulin* | 4 (1.6) |
| *Yes, I use somewhat less insulin* | 9 (4.0) |
| *No, I use the same amount of  insulin as before* | 177 (78.0) |
| *Yes, I use somewhat more insulin* | 33 (14.6) |
| *Yes, I use much more insulin* | 4 (1.6) |
| Do you manage to take your medication as usual during this period? N (%) | *N*=535 |
| *Yes, just as good as usual* | 484 (90.5) |
| *Yes, is going better than usual* | 29 (5.4) |
| *No, not as good as usual* | 22 (4.1) |
| Change in weight? n (%) | *N*=565 |
| *Yes, I lost ≥ 5 kilograms* | 15 (2.7) |
| *Yes, I lost 3-4 kilograms* | 30 (5.3) |
| *Yes, I lost 1-2 kilograms* | 52 (9.2) |
| *No, my weight remained the same* | 257 (45.5) |
| *Yes, I gained 1-2 kilograms* | 147 (26.0) |
| *Yes, I gained 3-4 kilograms* | 50 (8.8) |
| *Yes, I gained ≥ 5 kilograms* | 14 (2.5) |
| How often did you leave your house on an average day in the past 6 weeks? | *N*=565 |
| *I avoid going out of my house* | 129 (22.8) |
| *I only left my house for work* | 68 (12.0) |
| *1-2 times a week* | 253 (44.7) |
| *3-5 times a week* | 81 (14.3) |
| *>5 times a week* | 35 (6.2) |
